# The association between perceived discriminations and well-being in Korean employed workers: the 4th Korean working conditions survey

**DOI:** 10.1186/s40557-017-0205-9

**Published:** 2017-10-02

**Authors:** Hee Sung Lee, Guang Hwi Kim, Sung Won Jung, June-Hee Lee, Kyung-Jae Lee, Joo Ja Kim

**Affiliations:** Department of Occupational & Environmental Medicine, Soonchunhyang University Hospital, Seoul, South Korea

**Keywords:** Perceived discrimination, Well-being, Korean working conditions survey(KWCS), WHO-5 index

## Abstract

**Background:**

Around the globe, discrimination has emerged as a social issue requiring serious consideration. From the perspective of public health, the impact of discrimination on the health of affected individuals is a subject of great importance. On the other hand, subjective well-being is a key indicator of an individual’s physical, mental, and social health. The present study aims to analyze the relationship between Korean employed workers’ subjective health and their exposure to perceived discrimination.

**Methods:**

The Fourth Korean Working Conditions Survey (KWCS, 2014) was conducted on a representative sample of the economically active population aged 15 years or older, who were either employees or self-employed at the time of interview. After removing inconsistent data, 32,984 employed workers were examined in this study. The data included general and occupational characteristics, perceived discrimination, and well-being. Well-being was measured through the WHO-Five index (1998 version). Multiple logistic regression analysis was used to examine the association between perceived discrimination and well-being.

**Result:**

As a group, employed workers who were exposed to discrimination had a significantly higher likelihood of “poor well-being” than their counterparts who were not exposed to discrimination. More specifically, the workers exposed to age discrimination had an odds ratio(OR) of 1.51 (95% CI: 1.36–1.68), workers exposed to discrimination based on educational attainment had an OR of 1.43 (95% CI: 1.26–1.61), and workers exposed to discrimination based on employment type had an OR of 1.68 (95% CI: 1.48–1.91) with respect to poor well-being.

Furthermore, workers exposed to a greater number of discriminatory incidents were also at a higher risk of “poor well-being” than their counterparts who were exposed to fewer such incidents. More specifically, the workers with three exposures to discrimination had an OR of 2.60 (95% CI: 1.92–3.53), the workers with two such exposures had an OR of 1.69 (95% CI: 1.44–1.99), and the workers with one such exposure had an OR of 1.32 (95% CI: 1.20–1.45).

**Conclusion:**

The present study found that discrimination based on age, educational attainment, or employment type put workers at a higher risk of “poor well-being,” and that the greater the exposure to discrimination, the higher the risk of poor well-being.

## Background

Discrimination is defined as the unjust or prejudicial treatment of different categories of people in daily matters, including those pertaining to employment, on the grounds of characteristics determined at birth or by society [1]. Discrimination has emerged as a serious social problem in recent decades throughout the world. According to a 2004 Korean survey involving 2000 participants, 54.5% reported that discrimination was a serious social issue in Korea, and that discrimination against individuals with disabilities, low educational attainment, and immigrant workers, in particular, was the most rampant [1]. Aside from these, discrimination on the basis of one’s birthplace, socioeconomic class, employment type, and so on is also a social issue of concern that sparks discussions. Discrimination not only introduces conflict between members of society and feeds inequity, but also has a deleterious effect on the physical and mental health of individuals who are exposed to it [2, 3]. Ever since it has become clear that discrimination is a public health issue, a growing body of public health research has been dedicated to studying the impact of discrimination on the physical and mental health of individuals that it affects [2]. While early discrimination studies mostly focused on the impact of discrimination on mental health problems such as depression and stress, the scope of recent studies has expanded to its impact on health risk behaviors (tobacco and alcohol use), chronic diseases (metabolic syndrome, cardiovascular diseases), and systemic diseases (headache, myalgia, etc.) [3–6].

Well-being is a highly valued concept in modern society as the focus of its constituents has shifted from mere survival to a better life. Going beyond the simple absence of disease or infirmity, the concept of well-being encompasses life satisfaction characterized by positive emotions and unencumbered by negative emotions [7]. Therefore, well-being (WHO-5 Well-Being Index) is an effective indicator of an individual’s physical, mental, and social health [7].

Because it is widely known that well-being is closely associated with an individual’s mental health, depression screening tests often mention WHO-5 index scores [8]. Poor well-being has been associated with chronic diseases such as diabetes and cardiovascular diseases [9, 10]. Several studies have explained the link between well-being and physical health by demonstrating that individuals with robust well-being tend to have a stronger immune system and better physical health overall [11–13]. Studies have also presented a positive feedback loop between workers’ well-being and productivity, wherein workers with greater well-being also have a higher level of productivity, which in turn contributes to greater happiness [14].

For many individuals, the workplace is where they spend most of their daily hours and forge various interpersonal relationships. Consequently, it is also a place where individuals are exposed to various types of discrimination based on age, educational attainment, gender, birthplace, and so on [6]. From the perspective of public health, it is important to identify workplace discrimination in order to be able to prevent and manage discriminatory practices. A previous study reported that unfair treatment at a workplace on the basis of age, gender, and disability is associated with higher rates of absenteeism and self-reported diseases [6]. Other studies have reported that exposure to discrimination at work had a significant correlation with the poor self-rated health of workers and poor health outcomes [15, 16]. Studies examining discrimination among Korean employed workers are relatively scarce, and those investigating the various subtypes of discrimination are even fewer. The present study examines the types and frequency of workplace discrimination with the aim to identify their impact on Korean employed workers’ well-being.

## Methods

### Study population

The present study used data from the 2014 Korean Working Conditions Survey, the fourth survey of the Occupational Safety and Health Research Institute. Data were collected on employees older than 15 years of age. Trained interviewers collected data from each respondent. A total of 50,007 respondents met the criteria and were classified as employers, self-employed workers, paid workers, unpaid family workers, or others [17].

In this study, we defined the participants as only “employed workers aged over 19” in “companies with more than 2 employees”; therefore, we excluded “a self-employed worker” or “an unpaid worker in a family business.”(13,774workers).

Further, we also excluded individuals with incomplete responses(457workers), military personnel(78workers), and individuals who worked in the fishery and forestry industries(2,743workers). After excluding these individuals, we included 32,984 paid workers [18].

### WHO-five well-being index

Well-being was evaluated through the 5-item WHO-Five Well-Being Index (WHO-5), which is a short yet comprehensive tool used worldwide to measure subjective well-being [19]. The index consists of 5 positively phrased items designed to reflect whether well-being is present. As a self-reporting scale, respondents are asked to rate the positive emotions they felt over the last two-week period on a scale of 0 to 5. The total possible score range is from 0 to 25 points. In the present study, “poor well-being” was defined as a total score of less than 8 points, and “fair well-being” was defined as a minimum total score of 8 points [8, 20–26].

### General and occupational characteristics

The general characteristics of the respondents included were as follows: gender (male and female), age (20s, 30s, 40s, 50s, and 60s and older), educational attainment (lower than middle school degree, high school degree, associate’s degree, and university or higher), and monthly income (less than 1.3 million Korean won(KRW), 1.3 million – 2 million KRW, 2 million – 3 million KRW, and 3 million KRW and above) [17].

The occupational characteristics of the respondents included were as follows: occupation type (management/professional, office work, sales/service, simple labor), weekly working hours (less than 40 h per week, 41–52 h per week, 60 h per week or more), and employment type (permanent, temporary, daily) [27–29].

### Perceived discrimination

The item designed to assess perceived discrimination, “During the past 12 months, were you exposed to workplace discrimination?” had three sub-items of age, educational attainment, and employment type, each of which was answered as a yes or no. The potential range of exposures to such discrimination was defined from 0 to 3 [18].

### Data analysis

A chi-square test was performed in order to examine the distribution of respondents’ general and occupational characteristics according to the level of exposure to discrimination. Subsequently, the relationship between well-being and general and occupational characteristics was analyzed.

A final analysis was performed using perceived discrimination as the independent variable and well-being as the dependent variable. For this procedure, a multiple logistic regression analysis was performed while excluding the general and occupational characteristics, which were found to be significantly correlated with well-being in the results of the chi-square test, and those variables that existing studies have shown to be linked to well-being. In this study, the distribution of the well-being was overdispersed and zero-inflated. Thus we selected a zero-inflated negative binomial regression model to handle the distribution [30–32]. The model was used to analyse the association between perceived discrimination and well-being. Using this method, the incidence rate ratio(IRRs) and 95% CIs were calculated by adjusting all confounding variables. SPSS 24.0 was used for all statistical analyses.

## Results

### General and occupational characteristics of the study participants by type of perceived discrimination

More men than women comprised the 32,984 respondents that were examined in the present study. In terms of the age distribution, respondents in their 40s accounted for the largest share(29.3%), while those in their 60s and older accounted for the smallest share(11.3%). In terms of educational attainment, respondents with a university or higher accounted for the majority(38.2%), while those with a middle school degree or lower accounted for the smallest share(11.3%). In terms of employment type, office workers accounted for the largest share(28.5%), while simple laborers accounted for the smallest share(15.1%). In terms of income distribution, respondents with a monthly income of 2–3 million KRW accounted for the largest share(29.0%), while those with a monthly income of 1.3 million KRW accounted for the smallest share(20.5%). In terms of weekly working hours, respondents working 40 h per week or less accounted for the largest share(52.7%), while those working 61 h per week or more accounted for the smallest share(5.5%). In terms of employment type, permanent employees accounted for the largest share(76.5%), while daily contractors accounted for the smallest share(6.5%).

Exposure to age discrimination was greater among women than men, and the level of exposure increased with older age and lower educational attainment. All differences were found to be statistically significant. Simple laborers(10.9%) reported the highest level of exposure to age discrimination, which increased as income level decreased. The highest level of exposure to age discrimination was found among daily contractors(12.4%). All group differences were statistically significant.

Exposure to discrimination on the basis of educational attainment increased with decreasing age and increasing educational attainment, and all the differences were found to be statistically significant. Office workers (6.9%) and management/professionals (6.0%) reported high levels of exposure to education attainment discrimination, and the level of exposure increased as income level increased. The permanent employment type(5.5%) reported the highest level of exposure to educational discrimination. All group differences were statistically significant.

The respondents aged 60 years and older (5.3%), as well as the respondents in their 20s (5.2%) reported high levels of exposure to discrimination based on employment type, and the level of exposure increased as educational attainment decreased. All group differences were found to be significant. Further, simple laborers(6.8%) reported the highest level of exposure to such discrimination, which increased as income level decreased. The highest level of exposure to such discrimination was found among daily contract workers(9.2%), and all group differences were statistically significant (Table 1.).

**Table 1:** General and occupational characteristics of the study participants by type of perceived discrimination

	Total (*n* = 32,984)	Perceived discrimination								
		Age			Educational attainment			Employment type		
	N (%)	No (%)	Yes (%)	*P*-value*	No (%)	Yes (%)	P-value*	No (%)	Yes (%)	*P*-value*
General characteristics										
Gender										
Male	17,066 (51.7)	16,137 (94.6)	929 (5.4)	<0.001^†^	16,166 (94.7)	900 (5.3)	0.692	16,415 (96.2)	651 (3.8)	0.795
Female	15,918 (48.3)	14,869 (93.4)	1049 (6.6)		15,094 (94.8)	824 (5.2)		15,303 (96.1)	616 (3.9)	
Age (years)										
20–29	4331 (13.1)	4094 (94.5)	237 (5.5)	<0.001^†^	4018 (92.8)	314 (7.2)	<0.001^†^	4104 (94.8)	227 (5.2)	<0.001^†^
30–39	8624 (26.1)	8259 (95.8)	365 (4.2)		8113 (94.1)	511 (5.9)		8382 (97.2)	242 (2.8)	
40–49	9678 (29.3)	9199 (95.1)	479 (4.9)		9207 (95.1)	471 (4.9)		9370 (96.8)	308 (3.2)	
50–59	6622 (20.1)	6157 (93.0)	465 (7.0)		6331 (95.6)	291 (4.4)		6329 (95.6)	293 (4.4)	
≥ 60	3729 (11.3)	3296 (88.4)	433 (11.6)		3591 (96.3)	139 (3.7)		3533 (94.7)	196 (5.3)	
Education										
Middle school or lower	3739 (11.3)	3342 (89.4)	397 (10.6)	<0.001^†^	3589 (96.0)	149 (4.0)	<0.001^†^	3535 (94.5)	204 (5.5)	<0.001^†^
High school	11,975 (36.3)	11,173 (93.3)	802 (6.7)		11,429 (95.4)	546 (4.6)		11,415 (95.3)	559 (4.7)	
College	4686 (14.2)	4476 (95.5)	210 (4.5)		4449 (94.9)	237 (5.1)		4503 (96.1)	183 (3.9)	
University or higher	12,585 (38.2)	12,014 (95.5)	571 (4.5)		11,793 (93.7)	792 (6.3)		12,265 (97.5)	320 (2.5)	
Occupational characteristics										
Occupation type										
Management/professional	5358 (16.2)	5089 (95.0)	269 (5.0)	<0.001^†^	5035 (94.0)	323 (6.0)	<0.001^†^	5206 (97.1)	153 (2.9)	<0.001^†^
Office work	9412 (28.5)	9017 (95.8)	395 (4.2)		8765 (93.1)	647 (6.9)		9178 (97.5)	234 (2.5)	
Service/sales	8054 (24.4)	7547 (93.7)	507 (6.3)		7716 (95.8)	338 (4.2)		7742 (96.1)	312 (3.9)	
Technical	5180 (15.7)	4916 (94.9)	264 (5.1)		4972 (96.0)	208 (4.0)		4951 (95.6)	229 (4.4)	
Simple labor	4980 (15.1)	4436 (89.1)	544 (10.9)		4772 (95.8)	209 (4.2)		4641 (93.2)	339 (6.8)	
Monthly Income (10,000 won)										
< 130	6751 (20.5)	6150 (91.1)	601 (8.9)	<0.001^†^	6533 (96.8)	218 (3.2)	<0.001^†^	6370 (94.4)	381 (5.6)	<0.001^†^
130–199	8463 (25.7)	7857 (92.9)	605 (7.1)		7977 (94.3)	486 (5.7)		8056 (95.2)	407 (4.8)	
200–299	9576 (29.0)	9174 (95.8)	402 (4.2)		9081 (94.8)	495 (5.2)		9277 (96.9)	299 (3.1)	
≥ 300	8195 (24.8)	7824 (95.5)	371 (4.5)		7669 (93.6)	525 (6.4)		8016 (97.8)	179 (2.2)	
Working hours (per week)										
≤ 40	17,367 (52.7)	16,464 (94.8)	911 (5.2)	<0.001^†^	16,542 (95.2)	826 (4.8)	<0.001^†^	16,751 (96.5)	616 (3.5)	<0.001^†^
41–52	9627 (29.2)	9054 (94.0)	573 (6.0)		9060 (94.1)	567 (5.9)		9298 (96.6)	329 (3.4)	
53–60	4170 (12.6)	3860 (92.6)	310 (7.4)		3833 (94.3)	236 (5.7)		3967 (95.1)	203 (4.9)	
≥ 61	1820 (5.5)	1635 (89.8)	186 (10.2)		1725 (94.7)	96 (5.3)		1702 (93.5)	118 (6.5)	
Employment type										
Regular worker	25,231 (76.5)	23,936 (94.9)	1295 (5.1)	<0.001^†^	23,850 (94.5)	1381 (5.5)	0.001^†^	24,521 (97.2)	710 (2.8)	<0.001^†^
Temporary worker	5597 (17.0)	5180 (92.5)	417 (7.5)		5342 (95.4)	255 (4.6)		5239 (93.6)	358 (6.4)	
Day labor	2157 (6.5)	1889 (87.6)	267 (12.4)		2068 (95.9)	89 (4.1)		1958 (90.8)	199 (9.2)	

*Calculating using Chi-square test

^†^ *P* < 0.05

ªa 2–3 years course college

### Difference of general and occupational characteristics according to well-being

No significant gender difference was found in terms of “poor well-being” defined as a WHO-5 index score of 12 points or less. Prevalence of poor well-being increased with older age, lower educational attainment, lower income level, and longer working hours. All group differences were found to be statistically significant. Poor well-being was the most prevalent among simple laborers (29.8%) and daily contractors (29.7%), and the differences were statistically significant (Table 2.).

**Table 2:** Differences in general and occupational characteristics according to well-being

	Well-being		
	Fair	Poor	
	N (%)	N (%)	*P*-value*
General characteristics			
Gender			
Male	14,094 (82.6)	2972 (17.4)	0.097
Female	13,034 (81.9)	2884 (18.1)	
Age (years)			
20–29	3754 (86.7)	578 (13.3)	<0.001^†^
30–39	7451 (86.4)	1173 (13.6)	
40–49	7988 (82.5)	1690 (17.5)	
50–59	5269 (79.6)	1353 (20.4)	
≥ 60	2666 (71.5)	1063 (28.5)	
Education			
Middle school or below	2607 (69.7)	1132 (30.3)	<0.001^†^
High school	9564 (79.9)	2411 (20.1)	
College^b^	3956 (84.4)	730 (15.6)	
University or above	11,002 (87.4)	1583 (12.6)	
Occupational characteristics			
Occupation type			
Management/professional	4650 (86.8)	708 (13.2)	<0.001^†^
Office work	8194 (87.1)	1217 (12.9)	
Service/sales	6652 (82.6)	1401 (17.4)	
Technical	4137 (79.9)	1043 (20.1)	
Simple labor	3494 (70.2)	1486 (29.8)	
Monthly Income (10,000 won)			
< 130	5126 (75.9)	1625 (24.1)	<0.001^†^
130–199	6795 (80.3)	1668 (19.7)	
200–299	8077 (84.3)	1499 (15.7)	
≥ 300	7130 (87.0)	1065 (13.0)	
Working hours (per week)			
≤ 40	14,456 (83.2)	2911 (16.8)	<0.001^†^
41–52	8024 (83.3)	1603 (16.7)	
53–60	3291 (78.9)	879 (21.1)	
≥ 61	1357 (74.6)	463 (25.4)	
Employment type			
Regular worker	21,199 (84.0)	4032 (16.0)	<0.001^†^
Temporary worker	4413 (78.9)	1183 (21.1)	
Day labor	1516 (70.3)	641 (29.7)	

*Calculating using Chi-square test

^†^ *P* < 0.05

ªa 2–3 years course college

### Association between perceived discrimination and well-being

Regardless of the nature of discrimination (age, educational attainment, employment type), respondents who were exposed to workplace discrimination were found to have a higher incidence of poor well-being than their counterparts who were not exposed to such discrimination, and all differences were statistically significant. Furthermore, the highest incidence of poor well-being was found among the respondents who reported exposure to all three types of discrimination (35.1%). In fact, the incidence of poor well-being increased as the number of exposures to discrimination increased, and the differences were statistically significant (Table 3.).

**Table 3:** Level of well-being according to the type and number of exposures to workplace discrimination

Characteristics	Perceived discrimination	Well-being		
		Fair	Poor	
		N (%)	N (%)	*P*-value*
Age				
	No	25,686 (82.8)	5320 (17.2)	<0.001^†^
	Yes	1442 (72.9)	536 (27.1)	
Educational attainment				
	No	25,772 (82.4)	5488 (17.6)	<0.001^†^
	Yes	1356 (78.7)	368 (21.3)	
Employment type				
	No	26,226 (82.7)	5492 (17.3)	<0.001^†^
	Yes	903 (71.3)	364 (28.7)	
Numbers				
	0	24,246 (83.1)	4940 (16.9)	<0.001^†^
	1	2189 (77.6)	632 (22.4)	
	2	567 (72.4)	216 (27.6)	
	3	126 (64.9)	68 (35.1)	

*Calculating using Chi-square test

^†^P < 0.05

A multiple logistic regression analysis and zero-inflated negative binomial regression analysis were performed in order to produce the odds ratio and incidence rate ratio pertaining to well-being, discrimination type, and number of exposures. Age, education, occupation type, income, working hours, and employment type, all of which showed a significant correlation with the level of well-being, were excluded for this procedure.

Employed workers exposed to age discrimination, discrimination based on educational attainment, and discrimination based on employment type were found to be at significantly higher risks of “poor well-being” than their counterparts who were not exposed to such discrimination (OR 1.51, 95% CI: 1.36–1.68; OR 1.43, 95% CI: 1.26–1.61; OR 1.68, 95% CI: 1.48–1.91; and IRR 1.41, 95% CI: 1.28–1.56; IRR 1.37, 95% CI: 1.22–1.54; IRR 1.48, 95% CI: 1.31–1.67; respectively).

In terms of well-being according to the number of exposures to workplace discrimination, the risk of “poor well-being” increased significantly among the employed workers reporting one, two, and three exposures to workplace discrimination than their counterparts who reported zero exposure to workplace discrimination (OR 1.32, 95% CI: 1.20–1.45; OR 1.69, 95% CI: 1.44–1.99; OR 2.60, 95% CI: 1.92–3.53; and IRR 1.27, 95% CI: 1.16–1.40; IRR 1.54, 95% CI: 1.32–1.80; IRR 2.01, 95% CI: 1.52–2.67; respectively) (Table 4.).

**Table 4:** Odds ratios and incidence rate ratio for well-being according to the type and number of exposures to workplace discrimination

Characteristics	Perceived discrimination	OR^a^		IRR^b^	
		OR	95% CI	IRR	95% CI
Age					
	No	1.00	–	1.00	–
	Yes	1.51	1.36–1.68	1.41	1.28–1.56
Educational attainment					
	No	1.00	–	1.00	–
	Yes	1.43	1.26–1.61	1.37	1.22–1.54
Employment type					
	No	1.00	–	1.00	–
	Yes	1.68	1.48–1.91	1.48	1.31–1.67
Numbers					
	0	1.00	–	1.00	–
	1	1.32	1.20–1.45	1.27	1.16–1.40
	2	1.69	1.44–1.99	1.54	1.32–1.80
	3	2.60	1.92–3.53	2.01	1.52–2.67

^a^OR and 95% CI were calculated using multiple logistic regression model adjusted for sex, age, education, occupation type, income, working hours, and employment type

^b^IRR and 95% CI were calculated using a zero-inflated negative binomial regression model adjusted for sex, age, education, occupation type, income, working hours, and employment type

*OR* odds ratio, *IRR* incidence rate ratio, *CI* confidence interval

## Discussion

The present study utilized the Fourth Korean Working Conditions Survey data to examine the distribution of various types of workplace discrimination and identify their impact on Korean employed workers’ well-being. A multiple logistic regression analysis showed that discrimination based on age, educational attainment, and employment type was associated with employed workers’ well-being, and that the higher the exposure to these types of workplace discrimination, the stronger the association.

There was a significant gender disparity in exposure to age discrimination, which was reported by 5.4% of males and 6.6% of females. Although not presented in the current study, a significant gender disparity was found in terms of exposure to gender discrimination at work, with 0.9% of men and 3.0% of women reporting to have been exposed to such discrimination. This is consistent with a previous study, where female employed workers were found to be exposed to both gender discrimination and age discrimination [33].

When comparing exposures to discrimination according to age, significant differences were found across all types of exposures. Of these, workers in their 20s as well as workers in their 60s and older reported the highest level of exposure to discrimination based on employment type. Some studies have reported that the proportion of permanent employees decreased as workers’ age increased [27], and that temporary workers and daily contract workers tend to report a greater level of exposure to workplace discrimination based on employment type than their permanently employed counterparts [34]. The finding of the present study, wherein workers in their 60s and older had the lowest rate of permanent employment (40.4%), appears to be in line with the findings of previous studies.

Unlike other discrimination experiences, discrimination experiences according to educational attainment showed the highest rates in 2–30s, highly educated, white collar and management/ professional, high-earners, and regular workers, showing statistically significant differences. Although not represented in the table of this study, the proportion of young people with higher education in the 2–30 age group was high in white-collar and management/professionals, high-income earners and regular workers. In recent years, the inflation of educational attainment of the society, especially the 2–30 age group has become worse, and various problems related to employment and wages have arisen, and the employment or workplace competition among highly educated workers has been increasing recently [35]. For these complex reasons, we think that discrimination experiences according to educational attainment is high in 2–30s age group, highly educated, white collar, management/professionals, high income earners, and regular workers.

In the present study, women had higher rates of low educational attainment and non-regular employment (temporary or daily contract) than men. For this reason, it was expected that women would report a higher level of exposure to discrimination based on educational attainment or employment type. However, the gender difference was not statistically significant.

In the present study, the proportion of permanent employees in their 20s was the third lowest (60s and older: 40.4%, 20s: 74.1%). However, their exposure to discrimination based on employment type was the second highest, and on par with their counterparts aged 60 years and older. Although not represented in the current study, we obtained statistical results that the proportion of women in 20s, sales/service workers is high, and the proportion of high-income earners is low. Such outcomes are thought to be attributed to complex interactions of various factors such as gender, wage, and occupation type, in addition to employment type [36]. Several studies have examined older workers’ health and age discrimination at the workplace, but those concerning younger workers are currently lacking [3]. Therefore, it would be beneficial if more studies investigate the impact of workplace discrimination on the health of younger workers.

Group disparities in well-being according to age, educational attainment, occupation type, income, and employment type (factors other than gender and weekly working hours) were all statistically significant, a pattern that is similar to the findings of existing studies [37–39].

In terms of well-being and exposure to age discrimination, workers exposed to such discrimination had an OR of 1.51 (95% CI: 1.36–1.68) for poor well-being. Although age discrimination in a society typically pertains to discrimination against younger people, the opposite is true in a workplace, and this trend has been on the rise [40]. A classic example of age discrimination is including age limits in employment advertisements. Such age discrimination includes direct discrimination where candidates are unfairly treated because they are a certain age or fall into a certain age group, as well as indirect discrimination where candidates are initially assessed based on criteria other than age, but end up facing disadvantageous outcomes by way of falling into a certain age group [41]. Survey results show that a high percentage (58.6%) of Korean companies are reluctant to hire older workers, and that companies tend to use age as the basis for voluntary resignations and layoffs during employment adjustment [42]. Exposure to age discrimination in a workplace not only has a direct physical and psychological impact on older workers, but also can have an indirect health impact stemming from decreased wage, downward status adjustment in the job market, and loss of economic power [2]. The complex workings of these factors are thought to contribute to low well-being among workers discriminated against on the basis of age [21].

In terms of well-being and exposure to discrimination based on educational attainment, workers exposed to such discrimination had an OR of 1.43(95% CI: 1.26–1.61) for poor well-being. Educational attainment refers to the highest level of schooling that a person has completed. Academic cronyism is distinguished from educational attainment since it is an unofficial and underhanded concept that encourages a culture of faction, hierarchy, and superficiality wherein alumni network together to stay ahead [43]. Gaps in wage and employment are common outcomes of discrimination based on educational attainment and academic cronyism. According to a Korean survey, 64% of companies use candidates’ educational attainment and academic clique as a key index of employability [44]. According to another survey, for every 1.00 that workers with a high school degree earn, workers with a university earn 1.50, and workers with an associate’s degree earn 1.03 [45]. In modern society, discrimination based on educational attainment or academic clique is an increasing trend. In fact, it is a major source of complaints in terms of discrimination [15]. It is thought that workers exposed to such discrimination exhibit decreased well-being because of its direct negative impact on employment and its indirect negative impact on wage and other factors.

In terms of well-being and exposure to discrimination based on employment type, workers exposed to such discrimination had an OR of 1.68 (95% CI: 1.48–1.91) for poor well-being. In the present study, a high proportion of non-regular workers was found among respondents who are older, less educated, who are hired to perform simple labor, who are employed in sales/services, and who are in a low-income bracket. The findings are similar to those of existing studies that report that older, less educated, and low-income workers hired to perform high-risk tasks account for a high proportion of non-regular workers [46]. Another study reported that non-regular workers are more likely to be exposed to hazardous working conditions, and that they are not fairly compensated for the intensity and effort that the tasks require of them [47]. The study concludes that these workers have low levels of physical and mental health. There have also been studies that have reported that regularly employed workers report a higher level of self-related health than their non-regularly working counterparts [17]. In the present study as well, the groups with a large share of non-regular workers (old age, low education, low income, simple labor, sales/services groups) showed a low level of well-being, which is similar to the findings of existing studies [21].

Regarding well-being and the number of exposures to workplace discrimination, workers reporting one exposure had an OR of 1.32 (95% CI: 1.20–1.45) for poor well-being, while those reporting two exposures had an OR of 1.69 (95% CI: 1.44–1.99), and those reporting three exposures had an OR of 2.60 (95% CI: 1.92–3.53), indicating that the greater the exposure, the larger the OR and IRR for poor well-being.

The strengths of the current study are as follows: Firstly, it utilized national data to break down the types of discrimination that workers may be exposed to in a workplace and then analyzed their impact on workers’ well-being. Most existing studies examine the relationship between exposure to discrimination and psychological health by examining measures such as those for depression and insomnia. However, the present study verified the link between exposure to discrimination and well-being. Secondly, the effects of the specific nature of discrimination as well as the frequency of exposure to such discrimination on workers’ well-being were examined. Although studies that analyzed workers’ exposure to various types of discrimination already exist, the present study is the first to have both analyzed the types of discrimination and frequency of exposure in a workplace. The resulting findings show that the greater the number of exposures to discrimination, the greater the risk of poor well-being. This section summarizes the present study’s strengths.

On the other hand, the study also has some limitations. Firstly, it is a cross-sectional study, and thus, causality between discrimination and well-being cannot be confirmed. However, there are studies that have shown a link between exposure to discrimination and workers’ physical and mental well-being, as well as a link between well-being and health status. The current study’s outcomes are meaningful in that they appear to be connected to these findings [2, 15, 21]. Secondly, the questionnaire items surveying perceived discrimination are very subjective and simple, the types of discrimination they inquire about are not clearly explained, For example, the ratio of experience of age discrimination is high in daily workers (12.4%), and statistically significant is the fact that the proportion of women, aged, low education, low income and simple laborers is large in the distribution of daily workers as well as the age of daily workers, Likewise, it is thought that various complex causes have been acted upon.and there is a lack of structured items. However, studies continue to utilize the European EWCS or Korean KWCS to examine the relationship between exposure to inequality (including discrimination) and employed workers’ health [6, 48]. The current study is still relevant because its findings appear to be connected to those of previous studies. Third, we did not completely exclude the possibility of acting as a confounding variable of variables that are related to each discriminations experience. For example, the proportion of experience of age discrimination is the highest in daily labor workers (12.4%), and statistically significant is the fact that the proportion of women, aged, low education, low income and simple labor workers is large in the distribution of daily labor workers as well as the age of daily labor workers, likewise, it is thought that various complex causes have been acted upon. However, since it is not completely ruled out that the age has acted as a confounding variable, future studies should be supplemented.

Exposure to discrimination based on age, educational attainment, and employment type was analyzed in the current study. The odds ratios and incidence rate ratio were produced according to the number of personal exposures to such discrimination, and the results were significant. Exposure to discrimination based on race, gender, religion, disability, nationality, and so on could not be analyzed at this time due to an insufficient number of workers who had been exposed to discrimination of such origins. Follow-up studies examining a larger sample of employed workers, and that include and further analyze the workers’ individual characteristics, would be beneficial.

## Conclusion

The present study utilized data from the fourth Korean Working Conditions Survey to identify the relationship between employed workers’ well-being and exposure to various types of workplace discrimination. Exposure to discrimination based on age, educational attainment, and employment type put the workers at a higher risk of poor well-being, and the greater the exposure to discrimination, the higher the risk of poor well-being. As society’s interest in well-being continues to peak, identifying and preventing various types of workplace discrimination will greatly contribute to improving working conditions.

## Abbreviations

CI: Confidence interval
EWCS: European working conditions survey
IRR: Incidence rate ratio
KRW: Korean won
KWCS: Korean working conditions survey
OR: Odds ratio
WHO: World Health Organization
